# Uridine Triphosphate Thio Analogues Inhibit Platelet P2Y_12_ Receptor and Aggregation

**DOI:** 10.3390/ijms18020269

**Published:** 2017-01-29

**Authors:** Dursun Gündüz, Christian Tanislav, Daniel Sedding, Mariana Parahuleva, Sentot Santoso, Christian Troidl, Christian W. Hamm, Muhammad Aslam

**Affiliations:** 1 Department of Cardiology/Angiology, University Hospital Giessen, 35392 Giessen, Germany; dursun.guenduez@med.uni-giessen.de (D.G.); christian.troidl@med.uni-giessen.de (C.T.); christian.hamm@med.uni-giessen.de (C.W.H.); 2 Department of Neurology, University Hospital Giessen, 35392 Giessen, Germany; Christian.Tanislav@neuro.med.uni-giessen.de; 3 Department of Cardiology/Angiology, Hannover Medical School, 30625 Hannover, Germany; sedding.daniel@mh-hannover.de; 4 Department of Cardiology/Angiology, University Hospital Marburg, 35043 Marburg, Germany; mariana.parahuleva@innere.med.uni-giessen.de; 5 Institute for Clinical Immunology and Transfusion Medicine, Justus Liebig University, 35392 Giessen, Germany; sentot.santoso@med.uni-giessen.de

**Keywords:** 2*S*-UTP, 4*S*-UTP, P2Y_12_ receptor, ADP, platelet aggregation

## Abstract

Platelet P2Y_12_ is an important adenosine diphosphate (ADP) receptor that is involved in agonist-induced platelet aggregation and is a valuable target for the development of anti-platelet drugs. Here we characterise the effects of thio analogues of uridine triphosphate (UTP) on ADP-induced platelet aggregation. Using human platelet-rich plasma, we demonstrate that UTP inhibits P2Y_12_ but not P2Y_1_ receptors and antagonises 10 µM ADP-induced platelet aggregation in a concentration-dependent manner with an IC_50_ value of ~250 µM. An eight-fold higher platelet inhibitory activity was observed with a 2-thio analogue of UTP (2*S*-UTP), with an IC_50_ of 30 µM. The 4-thio analogue (4*S*-UTP) with an IC_50_ of 7.5 µM was 33-fold more effective. A three-fold decrease in inhibitory activity, however, was observed by introducing an isobutyl group at the 4*S*- position. A complete loss of inhibition was observed with thio-modification of the γ phosphate of the sugar moiety, which yields an enzymatically stable analogue. The interaction of UTP analogues with P2Y_12_ receptor was verified by P2Y_12_ receptor binding and cyclic AMP (cAMP) assays. These novel data demonstrate for the first time that 2- and 4-thio analogues of UTP are potent P2Y_12_ receptor antagonists that may be useful for therapeutic intervention.

## 1. Introduction

Platelets play a central role in vascular haemostasis. Uncontrolled platelet activation under certain pathological conditions may result in thrombus formation and occlusion of the vessels, leading to life-threatening cardiovascular events such as myocardial infarction [1,2] and thrombosis [3,4]. Platelets express nucleotide receptors P2Y_1_ and P2Y_12_ that are activated by adenosine diphosphate (ADP) and play a central role in platelet activation and aggregation [5,6,7,8]. Both P2Y_1_ and P2Y_12_ receptors are G-protein-coupled receptors that are coupled to G_q_ and G_i_, respectively [7]. P2Y_1_ receptor activation triggers Ca^2+^ mobilisation from the platelet dense tubular system and shape change [9,10], whereas activation of P2Y_12_ receptor causes an inhibition of adenylyl cyclase-dependent cyclic AMP (cAMP) production and platelet aggregation [7,11]. Because of its central role in thrombus formation and stabilisation, the P2Y_12_ receptor is a well-established target for anti-thrombotic drug development [12]. Presently, only a few P2Y_12_ receptor antagonists (both reversible and irreversible) are available for clinical interventions, and because of their pharmacokinetic profiles they have limitations in clinical application [12]. Therefore, there is a need for the development of novel P2Y_12_ receptor antagonists. Currently used P2Y_12_ receptor antagonists such as clopidogrel are either pro-drugs, requiring conversion to active metabolites by liver metabolic machinery, or are different nucleotide derivatives such as ticagrelor that reversibly bind to P2Y_12_ receptor and thus inhibit its activation. Uridine triphosphate (UTP) [13,14] and its thio-derivatives [15] act as natural and synthetic ligands for purinergic P2Y_2_ and P2Y_4_ receptors, respectively. The present study analysed the anti-platelet aggregation activity of UTP and a number of different thio-analogues, including UTPγS, aminoallyl UTP (AA-UTP), 2-thio UTP (2*S*-UTP), 4-thio UTP (4*S*-UTP), and 4-thio-isobutyl UTP (4*S*-*ib*-UTP). The study was carried out on platelet-rich plasma (PRP) isolated from freshly obtained blood from healthy human volunteers.

## 2. Results

### 2.1. Uridine Triphosphate (UTP) Thio-Analogues Antagonise Adenosine Diphosphate (ADP)-Induced Platelet Aggregation with Various Potencies

**Figure 1:**
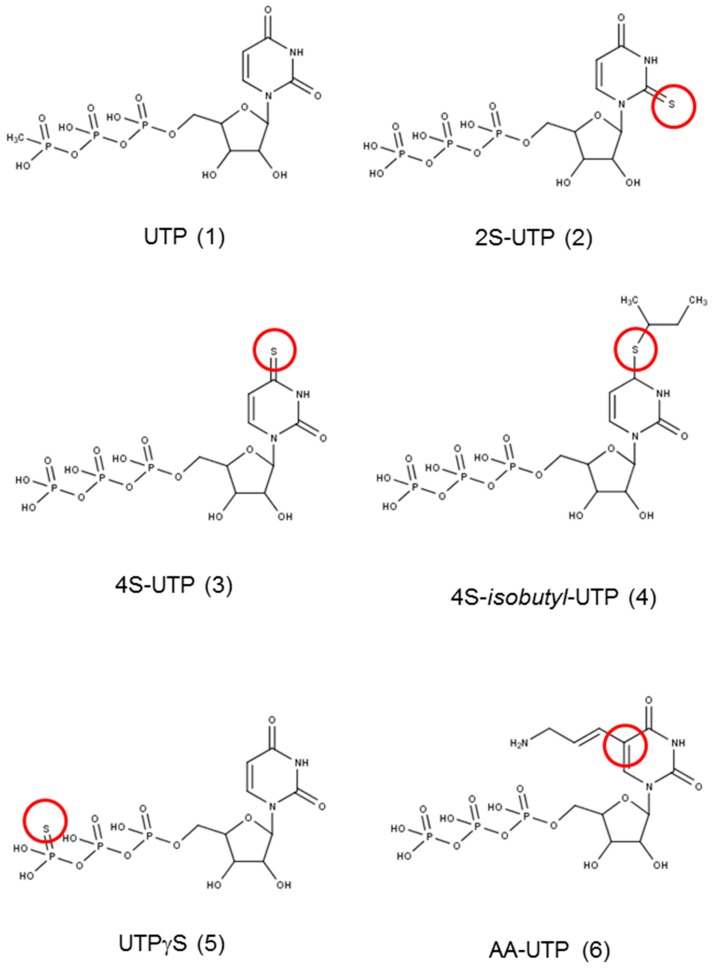
Structure of the compounds used in the study. The marked site shows the modification of the parent compound uridine triphosphate (UTP). Red circle mark the modified position in the original compound **1**.

**Figure 2:**
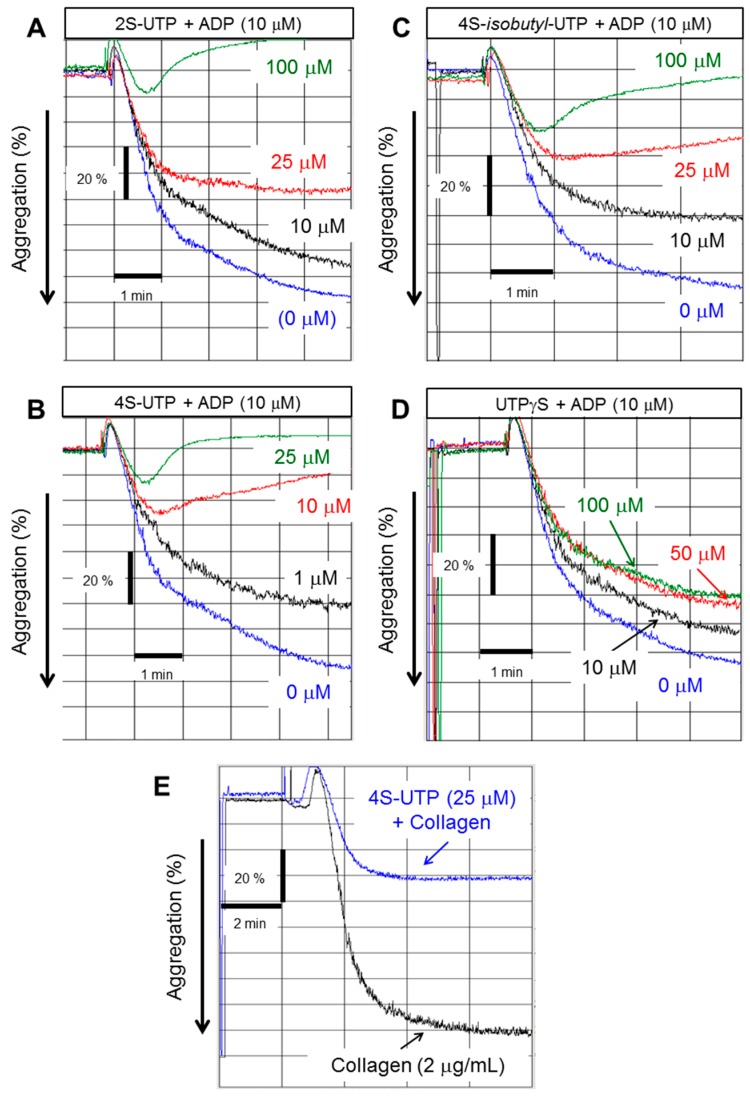
UTP thio-analogues antagonise adenosine diphosphate (ADP)-induced platelet aggregation. Representative tracing of platelet aggregation induced by ADP (10 µM) in the absence or presence of: 2*S*-UTP (**A**); 4*S*-UTP (**B**); 4*S*-isobutyl UTP (**C**); and UTPγS (**D**). Representative tracings from four experiments using independent PRP preparations. (**E**) Representative tracing of platelet aggregation induced by collagen (2 µg/mL) in the absence or presence of 4*S*-UTP (25 µM).

Figure 1 shows the structure of UTP and its analogues that were tested for anti-platelet aggregation activity in the present study. ADP at concentration of 10 µM caused 75%–90% platelet aggregation as measured by change in the turbidity of PRP (Figure 2). UTP antagonised ADP-induced platelet aggregation weakly with an IC_50_ value of ~250 µM (Table 1). The 2-thio derivative of UTP (2*S*-UTP; compound **2**) displayed a stronger inhibition of platelet aggregation (Figure 2A) with an IC_50_ of ~30 µM (Table 1). There was a 33-fold increase (vs. UTP) in platelet inhibition by thio (–*S*–) modification of –*O*– at position 4 of the pyrimidine ring. This compound (4*S*-UTP) was able to inhibit ADP-induced platelet aggregation completely at 15 µM (Figure 2B) with an IC_50_ of ~7.5 µM. Addition of an isobutyl group at the 4*S* position to yield 4*S*-*ib*-UTP, however, resulted in a three-fold decrease in inhibitory activity (Figure 2C). The non-hydrolysable stable analogue of UTP, UTPγS, showed very little activity against ADP-induced platelet aggregation (Figure 2D). Likewise, the aminoallyl derivative of UTP (AA-UTP) was virtually devoid of any anti-platelet activity. The IC_50_ values of these agents calculated from platelet aggregation data are given in Table 1. In addition to ADP, other agonists such as collagen cause the release of ADP from platelet dense granules thus indirectly activating P2Y_12_ receptor which is also involved in collagen-induced platelet aggregation. Therefore, it was investigated whether UTP analogues could also antagonise collagen-induced platelet aggregation. In this context experiments were performed only with the most active agent, 4*S*-UTP. Indeed 4*S*-UTP was able to strongly antagonise the collagen-induce platelet aggregation (Figure 2E).

### 2.2. Binding of UTP Thio-Analogues to P2Y_12_ Receptor, Cyclic AMP (cAMP) Production, and Vasodilator-Stimulated Phosphoprotein (VASP) Phosphorylation

**Figure 3:**
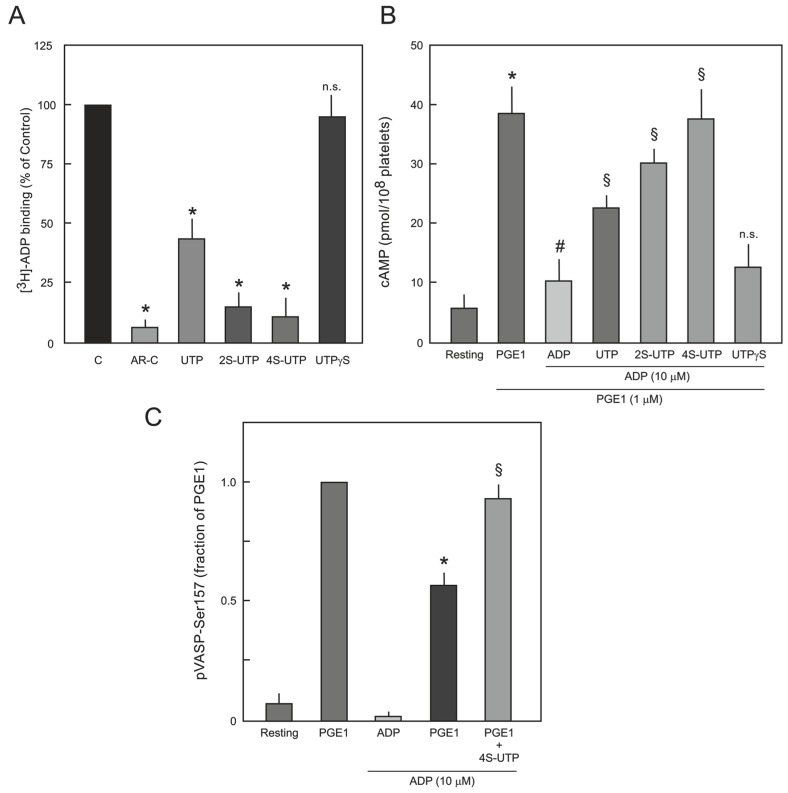
(**A**) Competitive binding of [^3^H]-ADP to platelet P2Y_12_ receptor. Platelets were incubated with 10 nM of [^3^H]-ADP in the absence (C; control) or presence of saturated concentrations (5x the respective IC_50_ values) of UTP, 2*S*-UTP, 4*S*-UTP, UTPγS and binding of [^3^H]-ADP to P2Y_12_ receptor was analysed as described in Section 4.4. AR-C66096 (AR-C; 10 µM; a potent P2Y_12_ receptor antagonist) was used as positive control. The data are means ± S.E.M of three experiments using independent platelet preparations. * *p* < 0.05 vs. control. n.s.: not significantly different from control; (**B**) Effect of UTP thio-analogues on cAMP level in PGE_1_- and ADP-stimulated human platelets. Different preparations of washed platelets were pre-incubated with PGE_1_ (1 µM) and UTP analogues (UTP 250 µM; 2*S*-UTP 100 µM; 4*S*-UTP 25 µM; and UTPγS 100 µM) for 10 min as indicated followed by stimulation with ADP (10 µM). * *p* < 0.05 vs. control, # *p* < 0.05 vs. PGE_1_, § *p* < 0.05 vs. ADP; (**C**) Effect of 4*S*-UTP on PGE_1_-mediated VASP phosphorylation (pVASP-Ser157) in ADP-stimulated human platelets. PRP was pre-incubated with vehicle (resting) or PGE_1_ (1 µM) followed by treatment with ADP (10 µM) or ADP plus 4*S*-UTP (25 µM) and pVASP-Ser157 was measured by flow cytometry. pVASP-Ser157 in the presence of PGE_1_-was taken as 1. * *p* < 0.05 vs. PGE_1_ alone, § *p* < 0.05 vs. PGE_1_ plus ADP.

ADP-induced platelet aggregation is mainly dependent on activation of platelet P2Y_12_ receptor. In order to confirm that UTP thio-analogue-mediated inhibition of ADP-induced platelet aggregation is due to antagonism at P2Y_12_ receptor, radioligand-displacement assays were performed using [^3^H]-ADP as the P2Y_12_ receptor agonist in the presence of P2Y_1_ antagonist (MRS2500 10 µM). As shown in Figure 3A, saturating concentrations of UTP thio-analogues competitively antagonised the binding of [^3^H]-ADP to P2Y_12_ receptor. 4*S*-UTP showed the strongest displacement of [^3^H]-ADP, and almost no displacement was observed with UTPγS (Figure 3A) and AA-UTP (data not shown).

Antagonism at P2Y_12_ receptor by UTP thio-analogues was further investigated by directly measuring cAMP production in platelets. Production of cAMP was induced by addition of prostaglandin E_1_ (PGE_1_; 1 µM), which was inhibited by the addition of ADP (10 µM). ADP-induced inhibition of cAMP was abrogated by UTP, 2*S*-UTP, and 4*S*-UTP but not by UTPγS (Figure 3B). Increased levels of cAMP induce phosphorylation of VASP at Ser157 via activation of protein kinase A (PKA) [16] and activation of P2Y_12_ receptor antagonises the PGE_1_-mediated VASP phosphorylation. Therefore, it was investigated whether UTP analogues could reverse ADP-induced reduction in PGE_1_-mediated VASP phosphorylation. In this context, experiments were performed only with the most active agent, 4*S*-UTP. Indeed, 4*S*-UTP could reverse ADP-induced reduction in PGE_1_-mediated VASP phosphorylation (Figure 3C).

### 2.3. Effect of UTP Thio-Analogues on ADP-Induced Platelet Shape Change

**Figure 4:**
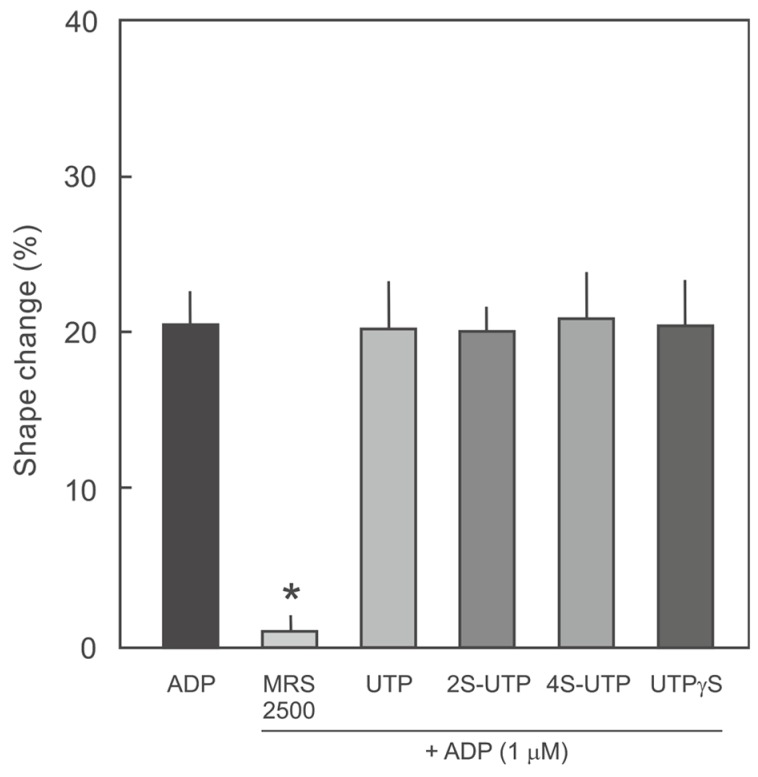
Effect of UTP thio-analogues on ADP-induced platelet shape change. Quantification of the platelet shape change data from three independent experiments. Platelet were pre-incubated with vehicle or MRS2500 (P2Y_1_ receptor antagonist; 1 µM), UTP (250 µM), 2*S*-UTP (100 µM), 4*S*-UTP (25 µM), or UTPγS (100 µM) and then treated with ADP (1 µM). * *p* < 0.05 vs. ADP alone.

Platelet P2Y_1_ receptor is responsible for ADP-induced platelet shape change and is partly involved in ADP-induced platelet aggregation. Therefore, we analysed whether UTP thio-analogues also inhibit P2Y_1_ receptor activation. ADP-induced platelet shape change was used as a measure of P2Y_1_ receptor activation. As shown in Figure 4, ADP (1 µM) induced a significant platelet shape change that was abrogated by the specific P2Y_1_ receptor antagonist MRS2500 but not by any of the UTP thio-analogues tested.

## 3. Discussion

The main and novel finding of the present study is that UTP thio-analogues 2*S*-UTP and 4*S*-UTP are potent antagonists of ADP- as well as collagen-induced platelet aggregation. The data from receptor binding and cAMP assays demonstrate that this inhibition is due to antagonism of P2Y_12_ receptors.

P2Y receptors are a class of purinergic G-protein-coupled receptors activated by naturally occurring extracellular nucleotides. In humans, eight P2Y receptors, namely P2Y_1_, P2Y_2_, P2Y_4_, P2Y_6_, and P2Y_11_–P2Y_14_, have been identified [17,18]. These P2Y receptors have variable affinity towards different natural nucleotides and nucleosides, e.g., ADP acts as a selective agonist for P2Y_1_ and P2Y_12_ receptors [19], but in contrast, ATP is a potent antagonist for both of these receptors [20,21] and an agonist for P2Y_2_ and P2Y_11_ receptors [22]. Similarly, UTP is a known natural agonist for P2Y_2_ and P2Y_4_ receptors [13,15], and UDP activates both P2Y_6_ and P2Y_14_ receptors [15,19].

Platelet aggregation is a complex process involving multiple receptors and signalling pathways. Platelet ADP receptors, particularly P2Y_12_ receptor, play a crucial role in agonist-induced platelet aggregation and thrombus formation [6,23]. Therefore, P2Y_12_ receptor is of particular interest in the quest to develop novel anti-thrombotic molecules for therapeutic interventions [12]. Activation of platelet P2Y_12_ receptor by ADP causes a reduction in platelet cAMP content, leading to platelet aggregation [7,21]. The data of the present study demonstrate that UTP derivatives, especially 2*S*- and 4*S*-UTP, potently antagonise ADP-induced reduction in cAMP and VASP phosphorylation, consistent with an inhibition of the P2Y_12_ receptor. This was further confirmed by P2Y_12_ receptor binding analyses. Activation of platelet P2Y_1_ receptor triggers Ca^2+^ mobilisation from the platelet dense tubular system and shape change [9,10]. Using platelet shape change as a measure of P2Y_1_ receptor activation we demonstrate that UTP and all of the analogues tested showed no antagonistic activity at this receptor.

Since nucleotides and nucleosides are natural ligands for purinergic receptors, structural modification of these compounds can be exploited to develop novel agonists and antagonists. For example, ADP is a natural ligand for P2Y_12_ receptor with an EC_50_ of 1.26 µM, and a thio-methyl (–SCH_3_) derivative of ADP (2MeSADP) is ~2000-fold more potent than ADP [24]. On the other hand ATP is an endogenous antagonist for P2Y_12_ receptor, and its thio-derivative is a highly potent P2Y_12_ receptor antagonist, cangrelor, that has recently been approved for clinical use [22]. UTP is a natural ligand for P2Y_2_ and P2Y_4_ receptors [13,15], and different derivatives of UTP have been developed to enhance the stability as well as affinity and specificity of the nucleotide towards these receptors. 2-Thio modification of UTP preserves its potency at the P2Y_2_ receptor and a further 2′-deoxy-2′-amino modification in the ribose ring further enhances potency and selectivity [17]. Similarly, thiol substitution of oxygen at position 4 resulted in a 4-fold increase in its agonist activity at the P2Y_2_ receptor [15]. In the present study, UTP was identified as a weak P2Y_12_ receptor antagonist; however, substitution of oxygen at either position 2 or 4 of the pyrimidine ring results in greatly enhanced antagonist activity at the P2Y_12_ receptor (with 4-thio substitution being more potent than 2-thio). Addition of a hydrophobic iso-butyl group to the 4–SH group, however, results in reduction in its activity. Addition of a –SH moiety to γ-phosphate of the sugar moiety results in an enzymatically stable compound [25] but this causes a strong reduction in its pEC_50_ from 8.10 (UTP) to 6.62 (UTPγS) as an agonist at the P2Y_2_ receptor [15,17]. Accordingly, UTPγS was devoid of antagonist activity at the P2Y_12_ receptor in the present study. Addition of an alkyl group at position 5 of the pyrimidine base resulted in reduction in its agonist activity at P2Y_2_ receptor [15]. This phenomenon was also observed in the present study, where addition of an amino-alkyl group at this position resulted in complete loss of its anti-platelet aggregation activity. Thiol modification of positions 2 and 4 of the pyrimidine ring in UTP possibly results in enhanced interaction of the nucleotide with cysteine residues of the P2Y_12_ receptor, resulting in enhanced antagonist activity. This assumption is based on previous studies demonstrating that active metabolites of both clopidogrel and prasugrel interact with extracellular cysteine residues of the P2Y_12_ receptor, which appears to be one the mechanisms of their receptor antagonism [26,27]. The present study is carried out on PRP, which although contains the major components of the platelet aggregation system but still lacks the cellular components of the blood. Therefore, IC_50_ values calculated in the PRP may differ from the whole blood aggregation studies.

## 4. Materials and Methods

### 4.1. Materials

PGE_1_ was from Biomol (Hamburg, Germany), anti-phospho-VASP (Ser157) antibody was from Cell Signaling (Danvers, MA, USA), Adenosine diphosphate (ADP) was from Enzo Life Science (Lörrach, Germany), 2-thiouridine 5′-triphosphate (2*S*-UTP), 4-thiouridine 5′-triphosphate (4*S*-UTP), 4-thio-isobutyl uridine 5′-triphosphate (4*S*-*ib*-UTP) was from Jena Bioscience (Jena, Germany), Collagen was from Nycomed (Linz, Austria), uridine triphosphate (UTP), UTPγS, and ARL 67156 were from Sigma (Steinheim, Germany). All other chemicals were of the best available quality, usually analytical grade.

### 4.2. Platelet-Rich Plasma (PRP) and Washed Platelet Preparation

The study conforms to the principles outlined in the “Declaration of Helsinki” (*Cardiovascular Research* 1997; 35: 2–3) for the use of human material. After approval from the local ethics committee of the University of Giessen (Giessen, Germany), peripheral blood was obtained from healthy human (male and female) volunteers (20–45 years old) who had not taken any drugs for at least 14 days. Blood samples were drawn into tubes containing trisodium citrate (Sarstedt, Germany). Whole blood was centrifuged at 110× *g* for 20 min at room temperature (RT) to obtain PRP. The platelet content was measured using an automatic haematology analyser Sysmex KX-21 (Sysmex, Germany). Platelet-poor plasma (PPP) was obtained by centrifugation of PRP at 14,000× *g* for 3 min. The platelet count in PRP was adjusted to (250–280) × 10^6^/mL by diluting native PRP with the same donor’s PPP.

In order to obtain washed platelets, the PRP was centrifuged at 600× *g* for 20 min at RT. The platelet pellet was re-suspended in Tyrode’s buffer (pH 7.2) containing PGI_2_ (0.5 µM) and albumin (0.1%) and the suspension was re-centrifuged at 600× *g* for 10 min. Finally, the washed platelets were re-suspended in Tyrode’s buffer (pH 7.2) at the concentration of 3 × 10^8^/mL. The suspended platelets showed a characteristic shimmering effect.

### 4.3. Platelet Aggregation and Shape Change

Platelet aggregation was measured with two-channel Chrono-Log aggregometer (Chrono-Log Corporation, Havertown, PA, USA) at 37 °C using stirred (1000 rpm) PRP. Various concentrations of agonists and/or antagonists as indicated in the figures or legends were added in a total volume of 50 µL NaCl (0.9%) solution for a final volume 500 µL. The relative platelet aggregation response to ADP was determined by comparison of light transmission through PPP (500 µL) and is expressed as a percentage response. UTP and its analogues were added 1 min prior to ADP addition.

The platelet shape change was measured using the offset mode of the Aggro/Link computer interface. The reference cuvette of the aggregometer contained a platelet suspension equivalent to 50% of the test samples (to amplify the signal). Abciximab (2 µg/mL) was added to all the samples to prevent platelet aggregation and obtain a stable shape change. The platelet shape change was monitored for 6–10 min after the addition of the agents.

### 4.4. [^3^H]-ADP P2Y_12_ Receptor Binding Assay

Binding of UTP analogues to P2Y_12_ receptor was determined by displacement of the binding of [^3^H]-ADP (PerkinElmer, Rodgau, Germany) to platelet P2Y_12_ receptor according to the protocol described by Savi et al. (2004) [28] using washed human platelets. Experiments were carried out in triplicate in a total volume of 100 µL Tyrode’s buffer (pH 7.2) containing 0.5 × 10^6^ platelets/µL and 10 nM [^3^H]-ADP at RT. The binding assays were performed in the presence of a P2Y_1_ receptor antagonist MRS2500 (10 µM). Non-specific binding was defined as the binding of [^3^H]-ADP measured in the presence of a saturating concentration of nonradioactive ADP (1 mM). For competitive binding of UTP analogues to P2Y_12_ receptor, the analogues were added at concentrations 5-fold higher than their respective IC_50_ values to obtain complete receptor saturation. The [^3^H]-ADP radioactivity was measured using an LS6500 (Beckman Coulter, Krefeld, Germany) automatic liquid scintillation counter.

### 4.5. Platelet cAMP Assay

cAMP levels were measured in washed platelets (100 µL) using a chemiluminescence-based HitHunter cAMP kit (DiscoveRx, Birmingham, UK) according to the manufacturer’s protocol. Briefly, washed platelets (100 µL) were incubated with prostaglandin E_1_ (PGE_1_, 1 µM) for 10 min in a 96-well plate. Subsequently, the platelets were stimulated with ADP (10 µM) in the absence or presence of UTP and its thio-analogues for 5 min. The reaction was stopped by adding stop buffer to the platelets and following the protocol outlined by the manufacturer. The cAMP content of each well was determined using an “Infinite^®^ 200” multi-plate reader (Tecan, Männedorf, Switzerland). Levels of cAMP were normalized to the platelet count used for the measurements.

### 4.6. Platelet VASP Phosphorylation

The platelet intracellular VASP-phosphorylation was determined by flow cytometry using anti-phospho-VASP (Ser157) antibody. PRP was incubated with vehicle or PGE_1_ (1 µM) for 10 min followed by the addition of ADP (10 µM) or 4*S*-UTP (25 µM) plus ADP and incubated for additional 5 min. Thereafter the platelets were fixed in 3% para formaldehyde for 10 min at room temperature (RT) and permeabilised with 0.2% triton X-100 for 10 min at RT and incubated with anti-phospho VASP antibody. After 30 min of incubation, platelets were washed in PBS and incubated with secondary FITC conjugated polyclonal anti-rabbit IgG antibody (DL488; Thermo fisher scientific, Dreieich, Germany) and analysed by FACS (Becton Dickinson, Heidelberg, Germany).

### 4.7. Data Presentation and Statistics

Each experiment was repeated at least 3 times. The data are presented as representative aggregometer tracings from a typical experiment. Dose–response curves were generated using Graphpad Prism software (Graphpad Software Inc., San Diego, CA, USA) from means ± S.E.M of transformed (i.e., percentage control) data pooled from 3 different experiments. The IC_50_ value for each agent was determined from 3 different concentrations of the agent using the Schild analysis function of Graphpad Prism.

## 5. Conclusions

UTP and its thio-derivatives are known to be potent agonists for P2Y_2_ and P2Y_4_ receptors [13,15]. Here, we describe for the first time that the thio-derivatives of UTP act as potent antagonists for platelet P2Y_12_ receptor. Addition of a thiol group at position 4 of the uracil moiety results in a highly potent P2Y_12_ receptor antagonist. Future studies exploiting modifications of this uracil position may provide even more potent reversible acting P2Y_12_ receptor antagonists that may be employed clinically to reduce thrombotic events.

## Figures and Tables

**Table 1:** IC_50_ values of UTP analogues.

Compound Number	Ligand	IC_50_ (µM)
1	UTP	250
2	2*S*-UTP	30
3	4*S*-UTP	7.5
4	4*S*-*ib*-UTP	23
5	UTPγS	>1000
6	AA-UTP	>1000

## Abbreviations

ATP	Adenosine 5′-triphosphate
ADP	Adenosine 5′-diphosphate
UTP	Uridine 5′-triphosphate
2*S*-UTP	2-Thiouridine 5′-triphosphate
4*S*-UTP	4-Thiouridine 5′-triphosphate
4*S*-*ib*-UTP	4-Thio-isobutyl uridine 5′-triphosphate
AA-UTP	5-Amino allyl uridine 5′-triphosphate

## Appendix A. Chemical Compounds Studied in This Article

ADP (PubChem CID: 6022); UTP (PubChem CID: 6133); 2*S*-UTP (PubChem CID: 10174453); 4*S*-UTP (PubChem CID: 3033941); 4*S*-*ib*-UTP (PubChem CID: Not available); AA-UTP (PubChem CID: 16218928); and UTPγS (PubChem CID: 5311494).
